# Impact of interval progression before autologous stem cell transplant in patients with multiple myeloma

**DOI:** 10.3389/fonc.2023.1216461

**Published:** 2023-07-24

**Authors:** Alicia Bao, Qiuhong Zhao, Ruchi Kudalkar, Jose Rodriguez, Nidhi Sharma, Naresh Bumma, Srinivas S. Devarakonda, Abdullah M. Khan, Elvira Umyarova, Ashley E. Rosko, Don Benson, Francesca Cottini

**Affiliations:** ^1^ The Ohio State University, College of Medicine, Columbus, OH, United States; ^2^ Department of Internal Medicine, Division of Hematology, College of Medicine, The Ohio State University, Columbus, OH, United States; ^3^ School of Medicine, Ponce Health Science University, Ponce, Puerto Rico

**Keywords:** myeloma, stem cell transplant (SCT), outcome, immune profiling, disease progression

## Abstract

In transplant-eligible patients who undergo upfront autologous stem cell transplant (ASCT) for multiple myeloma (MM), standard practice is to treat with six to eight cycles of induction therapy followed by high-dose chemotherapy with ASCT. A gap between the end of induction and the day of ASCT exists to allow stem cell mobilization and collection. Despite attempts to limit the length of this interval, we noticed that some patients experience interval progression (IP) of disease between the end of induction therapy and the day of ASCT. We analyzed 408 MM patients who underwent ASCT between 2011 and 2016. The median length of the interval between end of induction and ASCT was 38 days. We observed that 26% of patients in the entire cohort and 23.6% of patients who received induction with bortezomib-lenalidomide-dexamethasone (VRD) experienced IP. These patients deepened their responses with ASCT, independently of induction regimen. In the entire cohort, IP was significantly associated with shorter PFS in the univariable analysis (Hazard Ratio, HR = 1.37, *P* = 0.022) but not in the multivariable analysis (HR = 1.14, *P* = 0.44). However, analyzing only patients who received VRD as induction, progression-free survival (PFS) remained inferior in both the univariable (HR = 2.02; *P* = 0.002) and the multivariable analyses (HR = 1.96; *P* = 0.01). T cells and natural killer (NK) cells are increasingly studied targets of immunomodulatory therapy, as immune dysfunction is known to occur in patients with MM. Peripheral blood from 35 MM patients were analyzed. At time of ASCT, patients with IP had significantly increased percentages of CD3^+^CD8^+^CD57^+^ CD28^-^ (*P* = 0.05) and CD3^+^CD4^+^LAG3^+^ (*P* = 0.0022) T-cells, as well as less CD56^bright^ and CD56^dim^ NK cells bearing activated markers such as CD69, NKG2D, and CD226. These data suggest that IP can impact the length of response to ASCT; therefore, further studies on the management of these patients are needed.

## Highlights

• Patients treated with Bortezomib-lenalidomide-dexamethasone induction who experience interval progression between the end of induction and the day of transplant have shorter remissions in response to autologous stem cell transplant (ASCT).
• Patients with IP have more exhausted T cells and less activated NK cells at time of transplant.

## Introduction

Multiple myeloma (MM) is characterized by the abnormal growth of monoclonal plasma cells in the bone marrow, leading to bone lesions and kidney injury. MM cancer cells produce either a complete monoclonal (M) protein or free kappa or lambda light chains (FLC), which can be measured and used as markers of disease to evaluate responses to therapy and progression. Uniform response and relapse criteria have been developed by the International Myeloma Working Group (IMWG) to categorize patient responses to treatment as complete response (CR), very good partial response (VGPR), partial response (PR), minimal response (MR), and stable disease (SD) based on the percent reduction in M protein or FLC (1). Conversely, disease progression is defined as an increase of more than 25% from lowest response value or difference between involved and uninvolved FLC. More recently, the introduction of minimal residual disease (MRD) evaluation confirms the importance of achieving a deep response to therapy, even in patients in CR, to improve survival (2).

Induction therapy followed by high dose chemotherapy with autologous stem cell transplant rescue (HDT/ASCT) is still the backbone of MM treatment, providing progression-free survival (PFS) benefits compared to induction therapy alone (3). The current treatment timeline involves 4-6 cycles of induction therapy, stem cell mobilization and collection, followed by high-dose conditioning chemotherapy and stem cell rescue (**Supplementary Figure S1A**). Stem cell collection usually results in a gap between the end of induction therapy and HDT/ASCT, during which patients do not receive disease-directed therapy. During this time interval, some patients experience interval progression (IP) of their MM, often losing part of the initial obtained response. The association between a CR *after* HDT/ASCT and long-term survival has been well studied, with most retrospective analyses finding a significant association between CR and improved progression-free survival (PFS), overall survival (OS), or both (4–7). The relationship between *pre-*HDT/ASCT CR and survival is less clear; some data suggest a pre-HDT/ASCT CR is a prognostic factor for long-term survival (8), while other studies found a survival difference that was trending towards significance only with longer follow-up (9, 10). Currently no data are available regarding the post-HDT/ASCT survival outcomes associated with IP between end of induction and the day of transplant. Moreover, studies have shown that patients gain benefit from HDT/ASCT even if CR is not achieved pre-transplant (11), and thus many institutions proceed with HDT/ASCT after 4-6 cycles of induction therapy, regardless of the post-induction response.

The immune system is known to play an important role in intrinsic anti-tumoral immunity. Prior studies have demonstrated less robust immune cells in MM patients, reporting higher percentages of regulatory T cells (Treg) (12), terminally-differentiated, senescent T cells (CD28^-^CD57^+^), and exhausted T cells (lymphocyte activation gene-3 (LAG3), programmed cell death-1 (PD-1), cytotoxic T-lymphocyte–associated antigen 4 (CTLA-4)) (13, 14). Similarly, fewer natural killer (NK) cell activating receptors, such as Natural Killer group 2D (NKG2D), CD16, and NKp44 were found in MM patients (15). Although these data demonstrate differences in immune composition between healthy individuals and MM patients, no studies known to us have been done examining the variability in immune subpopulation among patients who experience progression of disease before ASCT. In this paper, we evaluate post-induction, pre- and post-transplant responses in patients who underwent upfront ASCT with a gap of less than 100 days from the end of induction. We specifically investigate the percentage and disease characteristics of patients who develop IP before ASCT and the relationship with post-transplant responses, PFS, and OS from day of ASCT. We also report the phenotype of their T and NK cell population prior to ASCT.

## Methods

### Patient selection

The patients described in this article were enrolled in a retrospective single-center study, approved by the Ohio State University Institutional Review Board (2021C0101) after providing written informed consent for the Ohio State University MM registry (OSU-10115) in accordance with the Declaration of Helsinki. A retrospective analysis of 482 patients who underwent ASCT at The Ohio State University Wexner Medical Center between 2011 and 2016 was completed.

### Clinical assessment

MM labs, including protein electrophoresis with immunofixation and FLC, were obtained at diagnosis, end of induction, and Day -2 of ASCT (**Supplementary Figure S1A**). IP was defined as a 25% increase in the index protein between the end of induction and Day -2 of ASCT. To exclude trivial changes in the M-protein or in the KLC/LLC, patients needed to have more than 100 mg/dL of M-protein or more than 100 mg/L of free light chains in the presence of stable renal function. Treatment response was defined by the IMWG criteria at the end of induction therapy (post-induction), at Day -2 of ASCT (pre-transplant), and 30-60 days post-ASCT (post-transplant). Fluorescence *In Situ* Hybridization (FISH) studies were performed at diagnosis. High-risk cytogenetic abnormalities (HRCA) were considered the presence of gain/amplification of chromosome arm 1q21 (1q21+), t(4;14), t(14;16), and deletion of chromosome 17p [del(17p)]. When two or three of these HRCAs were detected, patients were considered to have double-hit or triple-hit MM, respectively (16).

### Peripheral blood sample collection and cell isolation

Peripheral blood (PB) samples from 35 patients previously collected on Day -2 of ASCT were available for analysis. PB samples from 7 healthy donors (HD) were purchased from Versiti, Inc. Peripheral blood mononuclear cells (PBMC) were separated by density gradient centrifugation over Ficoll-Paque.

### Flow cytometry staining and analysis

Previously stored PBMC samples were thawed the day of staining. Viability was evaluated by Zombie-Aqua VioBlue dye staining. Cells were washed with centrifugation with room-temperature phosphate-buffered saline (PBS). After resuspending cells in PBS, flow antibodies were added (**Supplementary Table S1**). Samples were incubated at room temperature for 20 minutes in a dark environment. After a second wash with room-temperature PBS, cells were analyzed using an AttuneX machine. Post- acquisition analysis was performed using FlowJo software.

### Statistical analysis

Descriptive statistics (median and range, or frequency and percentage) were used to summarize patient demographics and disease characteristics. Wilcoxon rank-sum tests, chi-square tests or Fisher’s exact tests were used to compare distributions of characteristics between non-progressors (NP) patients and patients with IP. The primary clinical outcomes evaluated were PFS and OS from ASCT. PFS was defined as the time from ASCT until either disease progression or death, while OS was defined as the time from ASCT until death or last follow-up. Poisson regression models with robust variance were used to evaluate the associations between patients’ characteristics and IP. This analytic approach provides an unbiased estimate of the relative risk when the outcome is common (greater than 10%) (17). Cox proportional regression models were used for OS and PFS analysis. For each of the models above, univariable analysis (UVA) models were conducted first to evaluate the associations between individual risk factors and the study outcome. Factors with *P* values < 0.10 were further evaluated in a multivariable analysis (MVA) model to estimate its independent effect. Maintenance therapy was evaluated as a time dependent covariate in the model. Stata 16 (College Station, Texas) was used for all analyses and all tests were two-sided with significance level at 0.05. Immunophenotypic analysis was performed on either the percentage of specific cellular populations or the Mean Fluorescence Index (MFI). One-way ANOVA with Bonferroni’s multiple comparisons analysis was used to compared immunophenotypic data from healthy donors (HD), patients with IP, or NP.

## Results

### Patient characteristics

In our MM registry, we initially identified 482 patients who underwent upfront ASCT for MM between 2010-2016 and had staging labs at the defined time points (**Supplementary Figure S1A**). We then selected for patients who elected for upfront ASCT within a year from diagnosis (n = 455) and had a gap between end of therapy and day of transplant of less than 90 days (n = 408) (**Supplementary Figure S1B** and **Table 1**). Thirty-five of these patients had available PB prior to ASCT and were included in the immune profiling analysis. A separate analysis was performed in n = 165 patients who received bortezomib-lenalidomide-dexamethasone (VRD) as induction strategy. The initial cohort of patients included were primarily male (n = 251/408, 61.5%), non-Hispanic White (NHW) individuals (n = 359/408, 88.0%), with IgG disease (n = 228/408, 55.9%). MM International Staging System (ISS) stages at diagnosis were relatively equally represented. Cytogenetic analysis showed 93/348 (26.7%) patients had 1q21+, 50/348 (14.4%) patients had t(11;14), 29/348 (8.3%) patients had t(4;14), and 31/348 (8.9%) patients had del(17p). Based on cytogenetics, 98/348 (28.2%) of patients were classified as having high-risk disease, with 23/348 (6.6%) and 4/348 (1.1%) having double-hit or triple-hit MM, respectively. As induction therapy, 186/408 (45.5.%) patients received a doublet regimen with either bortezomib-dexamethasone or lenalidomide-dexamethasone, 165/408 (40.4%) received a triplet regimen with bortezomib-lenalidomide-dexamethasone, 45/408 (11.1%) received cyclophosphamide with bortezomib-dexamethasone, and the remaining 12/408 patients (3.0%) received other regimens. The median time between diagnosis and ASCT was 163 days (range: 84-361 days) and the median gap between end of induction and ASCT was 38 days (range: 12-90 days). Most of the patients (n = 350/408, 85.8%) received melphalan 200 mg/m^2^ as their conditioning regimen, while 58/408 (14.7%) received melphalan 140 mg/m^2^. The median number of infused cells was 4.11 CD34^+^ cells/kg (range: 1.99-17.49). 85.3% (n = 348/408) of patients underwent maintenance therapy following ASCT.

**Table 1:** Patient characteristics in all the analyzed patients.

Variable of interest		All (n = 408)	NP (n = 302)	IP (n = 106)	*P*
Age	Median (range)	58 (35-73)	58 (35-72)	60 (38-73)	0.08
Infused cells	Median (range)	4.11 (1.99-17.49)	4.03 (1.99-17.49)	4.43 (2.01-14.24)	0.049
Gender (no, %)	Male	251 (61.5)	187 (61.9)	64 (60.4)	0.78
	Female	157 (38.5)	115 (38.1)	42 (39.6)	
Race (no, %)	NHB	46 (11.3)	34 (11.3)	12 (11.3)	0.99
	NHW	359 (88.0)	266 (88.1)	93 (87.7)	
	Other	3 (0.7)	2 (0.7)	1 (0.9)	
MM Type (no, %)	IGA	87 (21.3)	69 (22.8)	18 (17.0)	0.35
	IGG	228 (55.9)	168 (55.6)	60 (56.6)	
	Light Chain disease	93 (22.8)	65 (21.5)	28 (26.4)	
MM ISS stage (no, %)	I	141 (39.4)	107 (40.2)	34 (37.0)	0.46
	II	112 (31.3)	78 (29.3)	34 (37.0)	
	III	105 (29.3)	81 (30.5)	24 (26.1)	
Induction regimen (no,%)	VRD VD or RD CyBorD Other	165 (40.4) 186 (45.5) 45 (11.1) 12 (3.0)	125 (41.4) 133 (44.0) 33 (10.9) 11 (3.6)	40 (37.7) 53 (50) 12 (18.9) 1 (0.9)	
Conditioning regimen (no,%)	Melphalan 140 (mg/m^2^)	58 (14.2)	43 (14.2)	15 (14.2)	0.98
	Melphalan 200 (mg/m^2^)	350 (85.8)	259 (85.8)	91 (85.8)	
Maintenance post-ASCT (no, %)	No	60 (14.7)	39 (15.6)	21 (19.8)	0.09
	Yes	348 (85.3)	263 (84.4)	85 (80.2)	
Cytogenetics (no, %)		N = 348	N = 262	N = 86	
1q21+	No	255 (73.3)	194 (74.0)	61 (70.9)	0.57
	Yes	93 (26.7)	68 (26.0)	25 (29.1)	
t(11;14)	No	298 (85.6)	228 (87.0)	70 (81.4)	0.20
	Yes	50 (14.4)	34 (13.0)	16 (18.6)	
t(4;14)	No	319 (91.7)	240 (91.6)	79 (91.9)	0.94
	Yes	29 (8.3)	222 (8.4)	7 (8.1)	
Del(17p)	No	317 (91.1)	241 (92.0)	76 (88.4)	0.31
	Yes	31 (8.9)	21 (8.0)	10 (11.6)	
High-Risk cytogenetic features	None One Hit Double Hit Triple Hit	216 (62.1) 105 (30.2) 23 (6.6) 4 (1.1)	165 (63.0) 78 (29.8) 16 (6.1) 3 (1.1)	51 (59.3) 27 (31.4) 7 (8.1) 1 (1.2)	0.84
Risk stratification	Standard Risk	250 (71.8)	186 (71.0)	64 (74.4)	0.54
	High risk	98 (28.2)	76 (29.0)	22 (25.6)	

MM, multiple myeloma; NHW, Non-Hispanic White; NHB, Non-Hispanic Black; ISS, International staging system; VRD, Bortezomib, lenalidomide, dexamethasone; VD, Bortezomib, dexamethasone; RD, Lenalidomide, dexamethasone; CyBorD, Cyclophosphamide, bortezomib, dexamethasone; ASCT, autologous stem cell transplant; P, p-value.

### Prevalence of interval progression and its role in MM disease response pre- and post-ASCT

One hundred and six (26%) patients experienced IP based on our definition criteria, i.e. a 25% increase of the index protein between the end of induction therapy and Day -2 of ASCT. The median time between diagnosis and ASCT was 159 days in non-progressor (NP) patients (range: 84-361 days) and 177 days in IP patients (range: 86-359, *P* = 0.007) and the median time between the end of induction and ASCT was 36 days in NP patients (range: 12-90 days), and 43 days in IP patients (range: 13-83 days, *P* = 0.002). No imbalance was noted in terms of dose of conditioning regimen with ASCT or maintenance therapy between the IP and NP groups.

**Figure 1:**
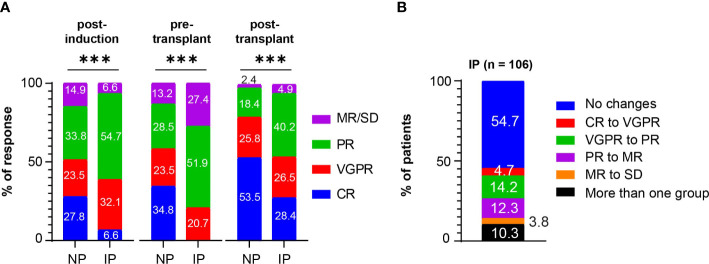
Post-induction, pre-transplant, and post-transplant responses in patients with MM. **(A)** Post-induction responses, pre-transplant responses, and post-transplant responses in non progressors (NP) or patients with interval progression (IP) are shown. Responses are presented as complete responses (CR), very good partial response (VGPR), partial response (PR), and minimal response or stable disease (MR/SD). Statistical test: Fisher exact test. P-values are as such: Post-induction *P* < 0.001, ***; pre-transplant *P* < 0.001, ***; post-transplant *P* < 0.001, ***. **(B)** The percentage of patients with interval progression (IP) (n = 106) who changed responses between end of induction and pre-transplant is shown. Responses are classified as in **(A).** Descriptive statistics is used in the analysis.

The extent of disease throughout induction and consolidation therapy was monitored using response classifications developed by the IMWG working group (**Figure 1A** and **Supplementary Table S2**). Among the NP group, 27.8%, 23.5%, 33.8%, and 14.9% were categorized as CR, VGPR, PR, and MR/SD/PD, respectively, in response to induction therapy. Among IP patients, 6.6%, 32.1%, 54.7%, and 6.6% achieved CR, VGPR, PR, and MR/SD, respectively, post-induction therapy (*P* < 0.001). As pre-transplant responses, the NP group had 34.8% CR, 23.5% VGPR, 28.5% PR, and 13.2% MR/SD, compared to 0% CR, 20.7% VGPR, 51.9% PR, and 27.4% MR/SD in the IP group (*P* < 0.001). As post-transplant responses, the NP group had 53.5% CR, 25.8% VGPR, 18.4% PR, and 2.3% MR/SD, compared to 28.4% CR, 26.5% VGPR, 40.2% PR, and 4.9% MR/SD in the IP group (*P* < 0.001). NP had more post-induction CR (CR: 27.8% versus 6.6%, *P* < 0.001), more post-transplant CR (CR: 53.5% versus 28.4%, *P* < 0.001) and less post-transplant PR (PR: 18.4% versus 40.2%, *P* < 0.001). The overall response rate (ORR) combines CR, VGPR, and PR percentages. Post-induction ORR, which includes CR, VGPR, and PR was higher in patients with IP, mainly driven by greater percentages of VGPR and PR (*P* = 0.03). As expected the pre-transplant ORR was in favor of patients without IP (*P* < 0.001), while the post-transplant ORR was not significantly different between the NP (97.7%) and IP (95.2%) groups (*P* = 0.19).

Among the patients with IP, 48/106 patients (45.3%) changed IMWG response groups between induction therapy and transplant, with 5 (4.7%) going from CR to VGPR, 15 (14.2%) going from VGPR to PR, 13 (12.3%) going from PR to MR, and 4 (3.8%) going from MR to SD, and the other 11 (10.3%) IP patients downgrading by more than one response group (**Figure 1B**). The remaining IP patients (n = 58, 54.7%) had at least a 25% increase of their MM markers but did not change response criteria group. Among the patients who were considered ORR to induction therapy (CR, VGPR, PR), 22 (20.3%) patients experienced IP that changed their classification to non-responsive (MR, SD, PD). Nearly all the patients in both the NP (95.2%) and IP (96.2%) groups at least maintained or deepened their responses after ASCT. However, 25/106 (23.6%) of patients with IP developed disease progression within 12 months post-transplant compared with 36/302 (11.9%) of NP (*P* = 0.007).

### Patient and disease variables associated with interval progression

UVA and MVA were performed to identify risk factors for IP (**Supplementary Table S3**). An increased risk of IP was associated with post-induction VGPR (RR = 2.41, 95% CI: 1.14-5.06, *P* = 0.02) or post-induction PR (RR = 2.69, 95% CI: 1.31-5.53, *P* = 0.01). No other patient or disease characteristics, such as gender, race, MM stage at diagnosis, chromosomal abnormalities, melphalan conditioning dose were identified. VGPR post-induction (RR = 2.37, 95% CI: 1.13-4.99, *P* = 0.02), and PR post-induction (RR 2.68, 95% CI: 1.30-5.52, *P* = 0.01) remained statistically significantly associated with increased risk of IP on MVA.

**Figure 2:**
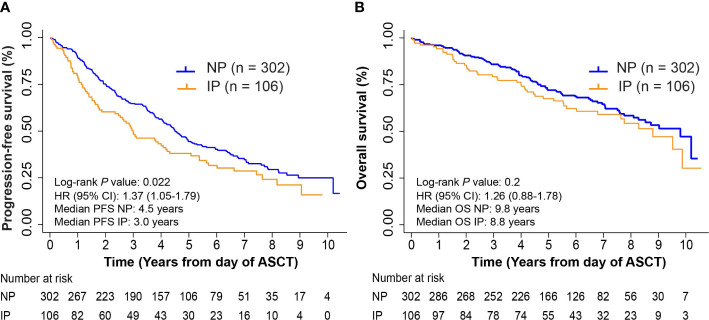
Progression-free survival (PFS) and overall survival (OS) in non-progressors (NP) and patients with interval progression (IP). **(A)** Kaplan-Meier survival curves for PFS from day of autologous stem cell transplant (ASCT). Log-rank *P* = 0.022. **(B)** Kaplan-Meier survival curves for OS from day of ASCT. Log-rank *P* = 0.2.

The overall median follow-up among those alive was 6.2 years from ASCT, 6.1 years for NP and 6.3 years for patients with IP, respectively. Kaplan-Meier survival analysis for PFS and OS from ASCT can be found in **Figures 2A, B**. IP was associated with inferior survival outcomes mainly in PFS (*P* = 0.022 and *P* = 0.2 for PFS and OS, respectively). Five-year PFS rates were 44.4% (95% CI: 38.5%-50.2%) and 38.1% (95% CI: 28.6%-47.4%) in the NP and IP groups, with median PFS of 4.5 (95% CI: 4.0-5.1 years) and 3.0 (95% CI: 2.3-4.2) years, respectively. Five-year OS rates were 72.1% (95% CI: 66.4%-76.9%) in the NP group and 67.5% (95% CI: 57.2%-75.9%) in the IP group, with median OS of 9.8 (95% CI: 8.1-not reached) and 8.8 (95% CI: 6.0-not reached) years, respectively. On UVA, IP correlated with inferior outcomes in PFS (Hazard Ratio, HR = 1.37, *P* = 0.022) but not in OS (HR = 1.26, *P* = 0.21) (**Supplementary Tables S4**, **S5**). Achieving CR post-induction (HR = 0.54, *P* = 0.004), CR pre-transplant (HR = 0.55, *P* = 0.002), or VGPR pre-transplant (HR = 0.62, *P* = 0.01) and the dose of infused cells (HR= 1.06, *P* = 0.03) were associated with improved PFS, but not OS (post-induction CR HR = 0.69, *P* = 0.162; pre-transplant CR HR = 0.70, *P* = 0.150; pre-transplant VGPR HR = 0.61, *P* = 0.06; Dose of infused cells HR = 1.06, *P* = 0.08). In agreement with previous literature, the following factors also independently correlated with inferior OS and PFS outcomes, including ISS stage III, cytogenetics (1q21+, t(4;14), and del(17p)), and absence of maintenance therapy post-ASCT (**Supplementary Tables S4**, **S5**). On MVA, IP was no longer significantly associated with a shorter PFS (HR = 1.14, *P* = 0.44), nor in conferring inferior OS (HR = 0.93, *P* = 0.72).

### Induction with bortezomib, lenalidomide, dexamethasone and its relationship with IP and outcomes

**Figure 3:**
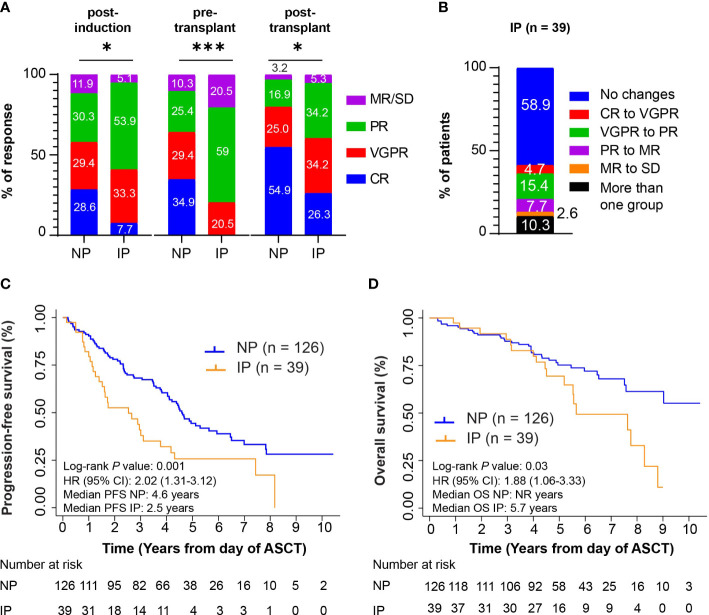
*Ad hoc* analysis in the cohort of patients who received VRD as induction strategy. **(A)** Post-induction responses, pre-transplant responses, and post-transplant responses in n = 126 non progressors (NP) or n = 39 patients with interval progression (IP) are shown. Responses are presented as complete responses (CR), very good partial response (VGPR), partial response (PR), and minimal response or stable disease (MR/SD). Statistical test: Fisher exact test. P-values are as such: Post-induction *P* = 0.01, *; pre-transplant *P* < 0.001, ***; post-transplant *P* = 0.01, *. **(B)** The percentage of patients with interval progression (IP) (n = 106) who changed responses between end of induction and pre-transplant is shown. Responses are classified as in **(A)** Descriptive statistics is used in the analysis. **(C)** Kaplan-Meier survival curves for PFS from day of autologous stem cell transplant (ASCT). Log-rank *P* value = 0.001. **(D)** Kaplan-Meier survival curves for OS from day of ASCT. Log-rank *P* value = 0.03.

To exclude that the above analysis may simply be reflective of suboptimal induction, we performed a separate analysis on 165 patients who received triplet-based induction therapy with VRD. The patient characteristics of this group were similar to the full cohort of patients (**Supplementary Table S6**). The majority of the patients received melphalan 200 mg/m^2^ as conditioning regimen (145/165, 87.9%) and underwent maintenance therapy (143/165, 86.7%). Among these patients, 39/165 (23.6%) experienced IP. The median time between diagnosis and ASCT was 161.5 days in NP patients (range: 97-343 days) and 170 days in IP patients (range: 100-340 days, *P* = 0.23), while the median time between the end of induction and ASCT was 34 days in NP patients (range: 12-85 days), and 37 days in patients with IP (range: 13-77 days, *P* = 0.120). The responses post-induction, pre-transplant, and post-transplant (**Figure 3A**) and the resulting changes in response categories (**Figure 3B**) were like those observed in the full cohort of patients. No patient, disease characteristics, or response to therapy were associated with IP. Also in this case, ASCT was associated with improved responses, with 24/39 (61.5%) patients with IP deepening their responses post-transplant. 8/39 (20.5%) patients with IP compared with 16/126 NP patients (12.7%) progressed within 12 months post-transplant (*P* = 0.29).

In this cohort of patients, inferior PFS and OS from ASCT were associated with IP (*P* = 0.001, and *P* = 0.03, **Figures 3C, D**). Five-year PFS rates were 44.4% (95% CI: 34.9%-53.4%) and 25.7% (95% CI: 12.7%-40.9%) in the NP and IP groups, with median PFS of 4.6 (95% CI: 4.0-5.6 years) and 2.5 (95% CI: 1.4-3.8) years, respectively. Five-year OS rates were 75.2% (95% CI: 66.0%-82.3%) in the NP group and 69.5% (95% CI: 50.3%-82.4%) in the IP group, with median OS of not reached (95% CI: 7.6-not reached) and 5.7 (95% CI: 5.2-8.3) years, respectively.

On UVA, IP correlated with inferior outcomes in PFS (HR = 2.02, *P* = 0.002) and in OS (HR = 1.88, *P* = 0.03) (**Supplementary Tables S7**, **S8**). As in the full cohort, ISS stage, cytogenetic risk, post-induction and pre-transplant responses, and dose of cells infused were associated with outcomes. On MVA, IP remained significantly associated with a shorter PFS (HR = 1.96, *P* = 0.01), but not with OS (HR = 1.61, *P* = 0.17).

### T and NK cell population immunophenotyping in NP or IP patients

PB samples from Day -2 of ASCT of 35 patients in the study were available for immunophenotypic analysis. Among them, we analyzed 13 patients with IP, 22 NP patients, and 7 healthy donors (HD) (**Supplementary Table S9**). This group of patients was representative of the cohort of all patients showing similar overall responses and inferior PFS from ASCT as in the full cohort (**Supplementary Figures S2A, B**).

PB was processed and stained for flow cytometry analysis as described in the methods section. The percentage of CD3^+^CD8^+^ cells was similar among the IP and NP groups (Mean subset cell percentage (MSCP): 22.34% in NP versus 28.11% in IP, *P* = 0.4056; HD: 39.81%; ANOVA summary *P* = 0.0025), with more CD3^+^CD8^+^ cells in NP compared with HD (*P* = 0.0018). The percentage of CD3^+^CD4^+^ cells (MSCP: 64.67% in NP versus 57.6% in IP, *P* = 0.7300; HD: 54.37%; ANOVA summary *P* = 0.2863), and the CD4**^+^**/CD8**^+^** ratio (3.5 in NP versus 3.0 in IP, *P* = 0.99; ANOVA summary *P* = 0.3425) were higher, though not significantly, in the NP group (**Supplementary Figures S3A, B**).

CD4^+^ and CD8^+^ T cells were then further characterized by their surface expression of CD45RA and CD62L to define antigen-exposed effector (Teff: CD45RA^+^CD62L^-^), effector memory (Tem: CD45RA^-^CD62L^-^), central memory (Tcm: CD45RA^-^CD62L^+^), and naïve T cells (Tn: CD45RA^+^CD62L^+^). Compared to HD, NP and patients with IP had less Tcm CD4^+^ cells (ANOVA Summary *P* = 0.0068), less Tnaive CD8^+^ cells (ANOVA Summary *P* = 0.0068) and less Tcm CD8^+^ cells (ANOVA Summary *P* = 0.0010). There was no statistically significant difference in CD8^+^ or CD4^+^ subtypes between the IP and NP groups (**Supplementary Figures S4A, B**).

**Figure 4:**
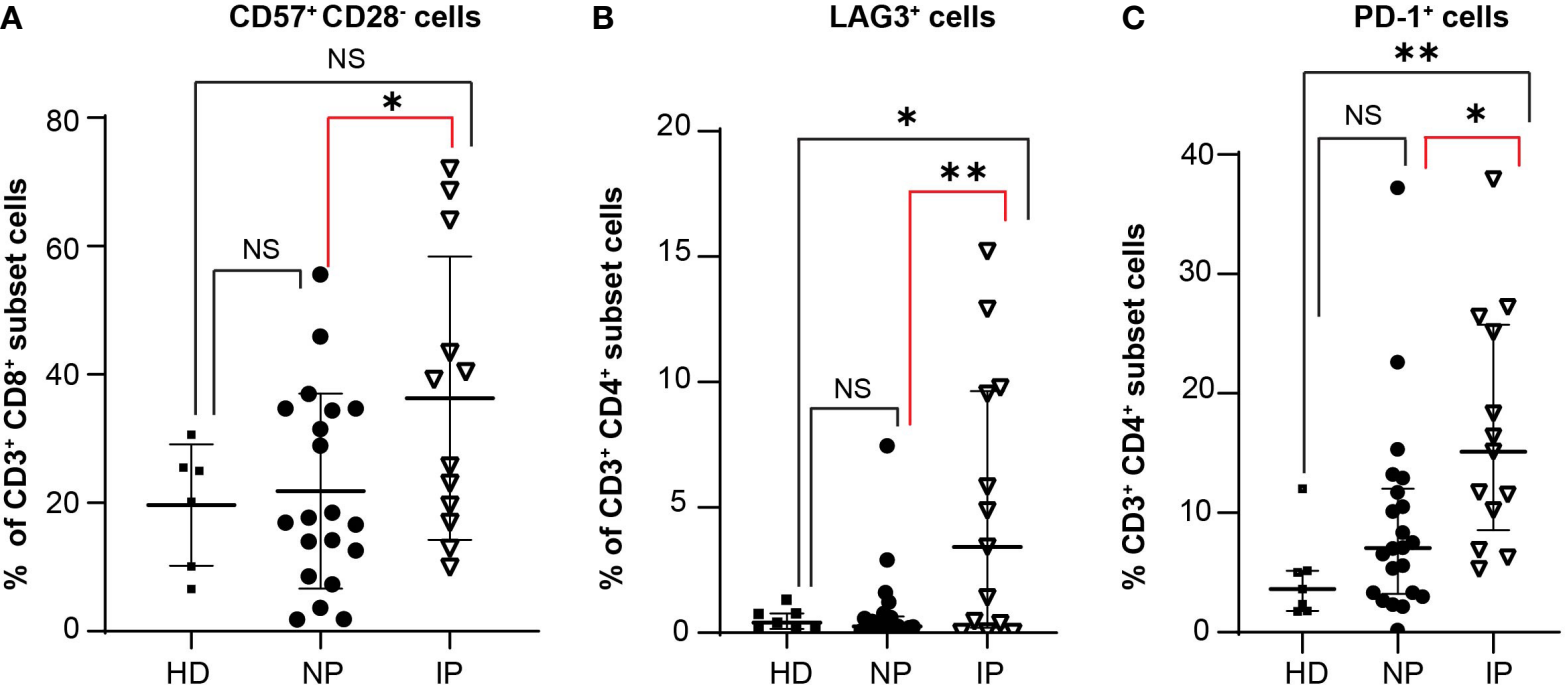
Increase in specific T cell populations in patients with interval progression (IP) compared with non-progressors (NP). The expression of the different markers is assessed in the peripheral blood of 7 healthy donors (HD), 22 NP, and 13 patients with IP. Percentages of **(A)** CD3^+^CD8^+^CD57^+^CD28^-^ T cells in HD, NP, and IP. One-way ANOVA with Bonferroni’s multiple comparison test: NS, HD versus NP; NS, HD versus IP; *, *P* = 0.05, NP versus IP; ANOVA summary *P* = 0.05. **(B)** CD3^+^CD4^+^LAG3^+^ T cells in HD, NP, and IP. One-way ANOVA with Bonferroni’s multiple comparison test: NS, HD versus NP; *, *P* = 0.017; HD versus IP; **, *P* = 0.0022, NP versus IP; ANOVA summary *P* = 0.0016. **(C)** CD3^+^CD4^+^PD-1^+^CD25^-^ T cells in HD, NP, and IP. One-way ANOVA with Bonferroni’s multiple comparison test: NS, HD versus NP; **, *P* = 0.0089, HD versus IP; *, *P* = 0.0305, NP versus IP; ANOVA summary *P* = 0.0055.

T cells express specific receptors, such as CD28, CD57, PD-1, or LAG-3, which are associated with different activation or inhibition status. We observed no statistically significant difference in the percentage of CD3^+^CD4^+^CD28^+^ T cells (MSCP: 17.19% in NP versus 28.8% in IP; *P =* 0.8535; HD: 20.37%; ANOVA Summary *P* = 0.5584) or CD3^+^CD8^+^CD28^+^ T cells (MSCP: 13.71% in NP versus 8.869% in IP; *P =* 0.99; HD: 21.94%; ANOVA Summary *P* = 0.2502) (**Supplementary Figure S5A**). Interestingly, patients with IP had higher percentages of CD3^+^CD8^+^CD57^+^CD28^-^ T cells (MSCP: 17.3% in NP versus 32.45% in IP, *P* = 0.05; HD: 22.6%; ANOVA Summary *P* = 0.05), which are considered senescent T cells (**Figure 4A**). Moreover, they also had higher percentages of CD3^+^CD4^+^LAG3^+^ T cells (MSCP: 0.81% in NP versus 4.910% in IP, *P* = 0.0022; HD: 0.53%; ANOVA Summary *P* = 0.0016), with greater, but not significant, LAG3 MFI values (98.22 in NP versus 121.4 in IP, *P* = 0.9; HD: 182; ANOVA Summary *P* = 0.1106) (**Figure 4B** and **Supplementary Figure S5B**). No statistically significant difference was noted in the percentage of CD3^+^CD8^+^LAG3^+^ T cells (**Supplementary Figure S5C**). The IP group also had a higher percentage of CD3^+^CD4^+^PD-1^+^CD25^-^ cells (MSCP: 8.9% in NP versus 16.78% in IP, *P* = 0.0305; HD: 4.51%, ANOVA Summary *P* = 0.0055) (**Figures 4C**). The percentage of CD3^+^CD8^+^PD1^+^CD25^-^ T cells did not differ between NP and IP (MSCP: 11.03% in NP versus 14.91% in IP, *P* = 0.56; HD: 4.76%, ANOVA Summary *P* = 0.0522), but patients with IP had more CD3^+^CD8^+^PD1^+^CD25^-^ T cells than HD (*P* = 0.0492) (**Supplementary Figure S5D**). (**Supplementary Figure S5D**). Finally, the percentages of Treg cells (CD3^+^CD4^+^CD25^+^ cells) did not differ between the two groups (MSCP: 4.4% in NP versus 3.971% in IP, *P* = 0.99; HD: 2.20%, ANOVA Summary *P* = 0.2442) (**Supplementary Figure S5E**).

**Figure 5:**
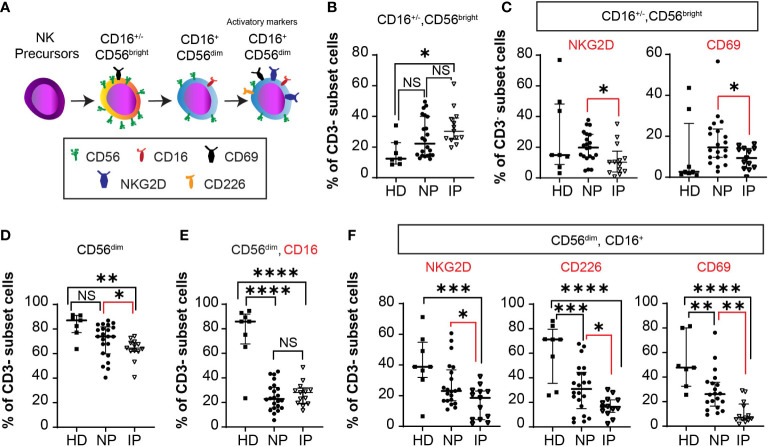
Accumulation of less mature NK cells in non-progressors (NP) compared with patients with interval progression (IP). **(A)** Schema of NK cell development with activation markers from NK precursors, to immature CD56^bright^CD16^+/-^ cells to mature CD56^dim^CD16^+^ cells, expressing activator markers. The expression of the different markers is assessed in the peripheral blood of 7 healthy donors (HD), 22 NP, and 13 patients with IP. Median with interquartile ranges are shown. Percentages of: **(B)** CD3^-^ CD56^bright^ CD16^-/+^ cells in HD, NP, and IP. One-way ANOVA with Bonferroni’s multiple comparison test: NS, HD versus NP; *, *P* = 0.0125, HD versus IP; NS, *P* = 0.23, NP versus IP; ANOVA Summary *P* = 0.0145. **(C)** CD3^-^ CD56^bright^ NKG2D^+^ or CD56^bright^CD69^+^ cells in HD, NP, and IP. One-way ANOVA with Bonferroni’s multiple comparison test: NKG2D: NS, HD versus NP; *, *P* = 0.0454, HD versus IP, *, *P* = 0.05, NP versus IP, ANOVA Summary *P* = 0.0452; CD69: NS, HD versus NP; NS, HD versus IP *, *P* = 0.031, NP versus IP; ANOVA Summary *P* = 0.13. **(D)** CD3^-^CD56^dim^ cells in HD, NP, and IP. One-way ANOVA with Bonferroni’s multiple comparison test: NS, *P* = 0.072, HD versus NP; **, *P* = 0.0034, HD versus IP; *, *P* = 0.049, NP versus IP. **(E)** CD3^-^CD56^dim^CD16^+^ cells in HD, NP, and IP. One-way ANOVA with Bonferroni’s multiple comparison test: ****, *P* < 0.0001, HD versus NP; ****, *P* < 0.0001, HD versus IP; NS, *P* = 0.99, NP versus IP; ANOVA Summary *P* < 0.0001. **(F)** CD3^-^ CD56^dim^ CD16^+^ NKG2D^+^ cells in HD, NP, and IP. One-way ANOVA with Bonferroni’s multiple comparison test: NS, HD versus NP; ***, *P* = 0.0009, HD versus IP; *, *P* = 0.046, NP versus IP; ANOVA Summary *P* = 0.0012; CD3^-^ CD56^dim^ CD16^+^ CD226^+^ cells in HD, NP, and IP. One-way ANOVA with Bonferroni’s multiple comparison test: ***, *P* = 0.0005, HD versus NP; ****, *P* < 0.0001, HD versus IP; *, *P* = 0.05, NP versus IP; ANOVA Summary *P* < 0.0001; and CD3^-^ CD56^dim^ CD16^+^ CD69^+^ cells in HD, NP, and IP. One-way ANOVA with Bonferroni’s multiple comparison test: **, *P* = 0.0052, HD versus NP; ****, *P* < 0.0001, HD versus IP; **, *P* = 0.0091, NP versus IP; ANOVA Summary *P* < 0.0001.

We then evaluated NK cell subsets in healthy donors and in the same two groups of patients as above. The different cellular subsets were gated as previously described (15). Total NK cells, defined as CD3^-^CD56^+^ cells, were distinguished by the relative expression of CD56 surface marker as either CD56^bright^ or CD56^dim^ cells. CD56^bright^ NK cells have low cytotoxicity activity, while CD56^dim^ NK cells are usually CD16^+^ and have more cytotoxic activity, representing an important source of NK-cell mediated anti-tumoral immunity. Together with CD16, DNAX accessory molecule 1 (DNAM1 or CD226), CD69, and NKG2D induce NK cell activation and enhance tumor lysis (**Figure 5A**). Patients with IP have more total (**Figure 5B**) immature CD56^bright^ NK cells (MSCP: 32.9% in IP versus 25.6% in NP, *P* = 0.23; HD: 16.4%; ANOVA Summary *P* = 0.0145). Moreover, these cells are less activated (**Figure 5C**) having less NKG2D or CD69 positive cells (NKG2D MSCP: 11.78% in IP versus 20.19% in NP, *P* = 0.05, HD: 28.1%; ANOVA Summary *P* = 0.0452; CD69 MSCP: 9.312% in IP versus 17.44% in NP, *P* = 0.031; HD: 11.47%; ANOVA Summary *P* = 0.13)

Patients with IP have less total CD56^dim^ cells (MSCP: 63.5% in IP versus 70.68% in NP, *P* = 0.049; HD: 82.99%; ANOVA Summary *P* = 0.046) compared with NP patients (**Figure 5D**). The percentage of CD56^dim^ CD16^+^ NK cells is lower in both IP and NP patients compared with HD (*P <*0.0001 and *P <*0.0001, respectively). Despite no statistically difference in the percentage of CD56^dim^ CD16^+^ NK cells between NP and IP patients (*P* = 0.99), **Figure 5E**, there was a lower percentage of CD56^dim^ CD16^+^ NKG2D^+^ cells (MSCP: 15.31% in IP versus 27.38% in NP, *P* = 0.046; HD: 41.0%; ANOVA Summary *P* = 0.0012), CD56^dim^ CD16^+^ CD226^+^ cells (MSCP: 16.59% in IP versus 31.54% in NP, *P* = 0.05; HD: 61.74%; ANOVA Summary *P* < 0.0001), and CD56^dim^ CD16^+^ CD69^+^ cells (MSCP: 11.06% IP versus 28.69% NP, *P* = 0.0091; HD: 51.83%; ANOVA Summary *P* < 0.0001) in patients with IP compared with NP (**Figure 5F**). All NK cell activation markers were lower in MM patients than in HD.

## Discussion

To produce a deep, sustained remission, transplant-eligible patients with MM receive six to eight cycles of induction therapy followed by melphalan conditioning therapy and ASCT, a strategy which ensures longer PFS compared to delayed transplant (3, 18, 19). By the nature of the treatment timeline, there is at least a month-long gap (median: 38 days in our full cohort; median: 35 days in patients treated with VRD induction) between the end of induction therapy and ASCT, during which patients are not receiving any disease-directed therapy.

CR or VGPR *post-*HDT/ASCT are known to be associated with improved survival outcomes (4–7), while the role of CR or VGPR post-induction or pre-ASCT is more controversial. Older studies using chemotherapeutic induction therapy demonstrated that patients who did not achieve CR post-induction still obtained a survival benefit from HDT/ASCT especially in PFS (8), while newer studies have mixed outcomes. Some suggest that depth of response at the end of induction or pre-ASCT still matters, recommending continuing induction therapy if CR is not obtained (20), while others focus on intensifying post-ASCT therapies with consolidation if no CR or MRD negativity is noted (21). This paper is the first to analyze long-term outcomes of patients who experienced progression of disease during the time gap between induction therapy and day of ASCT, an event not uncommon, as it occurred in a quarter of the patients of our database. In the entire cohort, IP was significantly associated with shorter PFS in the univariable analysis (HR = 1.37, *P* = 0.022) but not in the multivariable analysis (HR = 1.14, *P* = 0.44). However, analyzing only patients who received VRD as induction, progression-free survival (PFS) remained inferior in both the univariable (HR = 2.02; *P* = 0.002) and the multivariable analyses (HR = 1.96; *P* = 0.01). Our data also demonstrate that despite the clear short-term benefit of ASCT in patients with IP, they also tended to progress more commonly within the first 12 months from ASCT, a known poor prognostic feature in MM (22).

The presence of IP is also of additional significance because the collection of stem cells occurs during this time. A recent, prospective study showed MM cells in 40% of the collected stem cells, with the frequency of contamination and re-infusion directly correlating with the post-induction response and conferring a 2-fold risk increase in not achieving or delaying CR post-HDT/ASCT (23).

Immune cell dysfunction in patients with MM is common. ASCT tends to accelerate MM-associated T cell defects, with patients with high expression of LAG-3^+^ T cells having a shorter response to ASCT (24). In our study, we investigated T and NK cell phenotypes in samples obtained just prior ASCT in patients who were not receiving MM-directed therapy; therefore, the influence of anti-MM drugs on immune cells likely attenuated. We observed an increase in CD3^+^CD8^+^CD57^+^CD28^-^, CD3^+^CD4^+^LAG-3^+^ and CD3^+^CD4^+^PD-1^+^ T cells in patients with IP. Despite their efficacy in solid tumors, anti-PD-1 antibodies especially in combination with immunomodulatory drugs, were associated with adverse outcomes and toxicity in MM (25, 26). LAG-3 is another suitable target, with LAG-3 inhibitors increasing T cell function in *in vitro* studies, while currently being investigated in clinical trials (13). Similarly, reduced percentages of CD69^+^, CD226^+^, and NKG2D^+^ activated NK cells in both the CD56^bright^ and CD56^dim^ subpopulations were noted in patients with IP. Even though we do not have functional data on NK cells, the reduction of activated receptors would likely limit recognition of MM cells by NK cells, compromising anti-tumoral immunity in patients with rapidly growing disease (27).

This study has some limitations. Firstly, it is a single center retrospective study not performed in a controlled clinical setting, and as such, patients received a variable number of induction cycles as well as different regimens. As the trials demonstrating triplet therapy superiority were published more recently, patients who received older doublet regimens were included to permit analysis of long-term survival outcomes. However, 40.4% of patients received the now preferred triplet induction therapy and the analysis in this group confirmed reduced PFS from ASCT. Additionally, as this was the first project to study the progression of disease between end of induction and day of transplant, the definition of IP was arbitrarily set by this group, based on the IMWG definition of a 25% increase in M protein constituting progressive disease, in patients with stable kidney function and at least 100 mg/dL of free light chains or 0.1 g/dL of M-protein. Using a different percentage cutoff may yield variable survival results and may represent a future avenue of study. Another limitation is the relatively small number of peripheral blood samples available to study, which is though in line with previous studies (14, 15, 24). While the number of samples is limited, these samples are representative of the full cohort. Moreover, matched PB samples at MM diagnosis were not available, which could have been informative on the initial presentation of immune dysfunction in these patients. Nor were matched PB samples at first relapse obtained, which could have revealed data on clonal evolution or persistence of the immune composition.

In conclusion, we report that patients treated with VRD who experienced IP in the interval between induction therapy and ASCT have inferior PFS even in the MVA analysis, supporting the importance of achieving deep responses before proceeding to ASCT or other consolidation therapies to obtain PFS benefits. The management of patients with IP remains uncertain as it is unclear if they are destined to suboptimal outcomes independent of therapy or they can be salvaged with additional cycles of conventional therapy followed by ASCT or experimental strategies to modify their immune profiling. This study also confirms the fact that abnormal immune cell composition occurs early during the disease course and can potentially compromise future immunotherapeutic approaches. Therefore, early introduction of specific immunotherapy intervention, such as LAG-3 inhibition, might benefit patients with rapid disease progression or with an aggressive disease course.

## Data availability statement

The raw data supporting the conclusions of this article will be made available by the authors, without undue reservation.

## Ethics statement

The studies involving human participants were reviewed and approved by Ohio State University Institutional Review Board. Written informed consent for participation was not required for this study in accordance with the national legislation and the institutional requirements.

## Author contributions

All authors contributed to the article and approved the submitted version. AB performed research, collected, and analyzed data. QZ performed statistical analysis. RK and JR performed flow cytometry analysis of the peripheral blood of patients with MM and healthy individuals, NS collected retrospective MM patient data, NB, SD, AK, EU, AR, and DB consented patients and provided critical review of manuscript. FC performed and supervised data collection and analysis, wrote the IRB protocol and manuscript, supervised the study, and obtained funding.
