# Supplementary material for: Impact of interval progression before autologous stem cell transplant in patients with multiple myeloma
Source: Front Oncol. 2023 Jul 24;13:1216461. doi: 10.3389/fonc.2023.1216461 (PMC10405820; doi:10.3389/fonc.2023.1216461)
Supplement: Supplementary file 1 [file DataSheet_1.pdf]

Supplementary Contents:

Supplementary Table S1. Specific recognized antigens, clones, fluorochromes, suppliers, and catalogue numbers of flow cytometry antibodies used in the peripheral blood analysis.

Supplementary Table S2. MM responses at different time points during treatment.

Supplementary Table S3. Regression analysis for factors associated with interval progression (IP).

Supplementary Table S4. Regression analysis for factors associated with progression-free survival (PFS) in all patients.

Supplementary Table S5. Regression analysis for factors associated with overall survival (OS) in all patients.

Supplementary Table S6. Patient characteristics in the cohort of patients treated with VRD induction.

Supplementary Table S7. Regression analysis for factors associated with progression-free survival (PFS) in patients treated with VRD induction.

Supplementary Table S8. Regression analysis for factors associated with overall survival (OS) in patients treated with VRD induction.

Supplementary Table S9. Patient characteristics of samples from non-progressors and patients with interval progression used for flow cytometry analysis.

Supplementary Figure S1. Timeline of data collection.

Supplementary Figure S2. Patients with available samples for flow cytometry staining are representative of the entire cohort.

Supplementary Figure S3. No changes in total CD3+CD4+ T cells, CD3+CD8+ T cells, or CD4+/CD8+ ratios in non-progressors (NP) compared to patients with interval progression (IP)

Supplementary Figure S4. No changes in activation status of CD4+ and CD8+ T cell populations in non-progressors (NP) compared to patients with interval progression (IP)

Supplementary Figure S5. No changes in the percentage or Mean Fluorescence Intensity (MFI) values of other T cell populations in non-progressors (NP) compared to patients with interval progression (IP).

Supplementary Table S1. Specific recognized antigens, clones, fluorochromes, suppliers, and catalogue numbers of flow cytometry antibodies used in the peripheral blood analysis.

**Antigen**	**Clone**	**Fluorophore**	**Supplier**	**Catalogue number**
CD3	BW264/56	VioBlue	Miltenyi	170-081-046
CD4	RPA-T4 (RUO)	FITC	BD Bioscience	555346
CD8	BW135/80	APC	Miltenyi	130-113-154
LAG3/CD223	REA351	PE	Miltenyi	130-120-470
PD1/CD279	MIH4	PE	BD Bioscience	557946
CD28	15E8	PE	Miltenyi	130-126-172
CD25	M-A251	APC	BD Bioscience	555434
CD57	NK-1	FITC	BD Bioscience	555619
CD45RA	HI100	PE	BD Bioscience	561883
CD62L	145/15	VioBlue	Miltenyi	130-114-148
CD16	3G8	FITC	BD Bioscience	555406
CD56	REA196	APC Vio770	Miltenyi	130-114-548
CD56	B159	APC	BD Bioscience	555518
CD94	HP-3D9	PE	BD Bioscience	555889
NKG2A/CD159a	REA110	APC	Miltenyi	130-113-563
CD226	DX11	PE	BD Bioscience	559789
CD69	FN50	PE	Miltenyi	130-113-524
NKG2D/CD314	1D11	PE	BD Bioscience	557940

Supplementary Table S2. MM responses at different time points during treatment.

Variable of interest		NP (n = 302)	IP (n = 106)	*P*
**Post-induction response**	CR/VGPR/PR	257 (85.1)	99 (93.4)	0.03
	SD/MR/PD	45 (14.9)	7 (6.6)	
	CR	84 (27.8)	7 (6.6)	< 0.001
	VGPR	71 (23.5)	34 (32.1)	
	PR	102 (33.8)	58 (54.7)	
	SD/MR/PD	45 (14.9)	7 (6.6)	
**Pre-transplant response**	CR/VGPR/PR	262 (86.8)	77 (72.5)	<0.001
	SD/MR/PD	40 (13.2)	29 (27.5)	
	CR	105 (34.8)	0 (0.0)	<0.001
	VGPR	71 (23.5)	22 (20.7)	
	PR	86 (28.5)	55 (51.9)	
	SD/MR/PD	40 (13.2)	29 (27.4)	
**Post-transplant response**	CR/VGPR/PR	292 (97.7)	97 (95.1)	0.19
	SD/MR/PD	7 (2.3)	5 (4.9)	
	CR	160 (53.5)	29 (28.4)	<0.001
	VGPR	77 (25.8)	27 (26.5)	
	PR	55 (18.4)	41 (40.2)	
	SD/MR/PD	7 (2.3)	5 (4.9)	

Abbreviations: MM - multiple myeloma, CR - complete response, VGPR - very good partial response, PR - partial response, MR - minimal response, SD - stable disease, *P* – p-value.

Supplementary Table S3. Regression analysis for factors associated with interval progression (IP).

Factors		Univariable analysis				Multivariable analysis			
		**RR**	**95% CI**		***P***	**RR**	**95% CI**		***P***
	**Age at diagnosis**	1.02	0.99	1.04	0.17				
	**Dose of infused cells**	1.06	1.00	1.14	0.07	1.06	0.99	1.13	0.08
**Gender**	Male	1.00							
	Female	1.05	0.75	1.47	0.78				
**Race**	Non-Hispanic White	1.00							
	Non-Hispanic Black	1.01	0.60	1.69	0.98				
	Other	1.29	0.26	6.45	0.76				
**MM type**	IGA	1.00							
	IGG	1.27	0.80	2.03	0.31				
	Light chain disease	1.46	0.87	2.44	0.15				
**MM ISS stage**	I	1.00							
	II	1.26	0.84	1.89	0.27				
	III	0.95	0.60	1.50	0.82				
**Post-induction response**	CR/VGPR/PR	2.07	1.02	4.20	0.045				
	SD/MR/PD	1.00							
	CR	0.57	0.21	1.54	0.269	0.57	0.21	1.53	0.264
	VGPR	2.41	1.14	5.06	0.02	2.37	1.13	4.99	0.02
	PR	2.69	1.31	5.53	0.01	2.68	1.30	5.52	0.01
	SD/MR/PD	1.00				1.00			
**1q21+**	Yes	1.12	0.75	1.86	0.57				
	No	1.00							
**t(11;14)**	Yes	1.36	0.87	2.14	0.18				
	No	1.00							
**t(4;14)**	Yes	0.97	0.50	1.91	0.94				
	No	1.00							
**Del(17p)**	Yes	1.35	0.78	2.33	0.29				
	No	1.00							
**Risk stratification**	Standard risk	1.00							
	High risk	0.88	0.57	1.34	0.55				

Abbreviations: RR - relative risk, CI - confidence interval, MM - multiple myeloma, ISS - International staging system, CR - complete response, VGPR - very good partial response, PR - partial response, MR - minimal response, SD - stable disease, *P* – p-value.

Supplementary Table S4. Regression analysis for factors associated with progression-free survival (PFS).

Factors		Univariable analysis				Multivariable analysis			
		**HR**	**95% CI**		***P***	**RR**	**95% CI**		***P***
	**IP**	1.37	1.05	1.79	0.0022	1.14	0.82	1.59	0.44
	**Age at diagnosis**	1.00	0.99	1.02	0.90				
	**Number of cells infused**	1.06	1.01	1.12	0.03	1.02	0.96	1.08	0.61
Gender	Male	1.00							
	Female	1.00	0.78	1.28	0.99				
Race	Non-Hispanic White	1.00							
	Non-Hispanic Black	0.77	0.51	1.15	0.20				
	Other	0.50	0.07	3.55	0.49				
MM type	IGA	1.00				1.00			
	IGG	0.75	0.55	1.011	0.06	0.76	0.52	1.09	0.14
	Light Chain disease	0.79	0.55	1.13	0.20	0.64	0.41	1.01	0.06
MM ISS stage	I	1.00				1.00			
	II	1.05	0.77	1.44	0.75	0.93	0.66	1.33	0.71
	III	1.49	1.09	2.03	0.01	1.43	1.03	2.00	0.03
Post-induction response	CR	0.54	0.35	0.82	0.004				
	VGPR	0.76	0.51	1.12	0.17				
	PR	0.75	0.52	1.08	0.12				
	SD/MR/PD	1.00							
Pre-transplant response	CR	0.55	0.38	0.80	0.002				
	VGPR	0.62	0.43	0.90	0.01				
	PR	0.82	0.59	1.14	0.24				
	SD/MR/PD	1.00							
Conditioning regimen	Melphalan 140 (mg/m^2)	1.00							
	Melphalan 200 (mg/m^2)	0.89	0.63	1.26	0.51				
Maintenance post-transplant	No	1.00				1.00			
	Yes	0.39	0.30	0.50	<0.001	0.36	0.27	0.49	<0.001
**Cytogenetics**									
1q21+	Yes	1.63	1.23	2.17	0.001	1.66	1.21	2.28	0.002
	No	1.00							
t(11;14)	Yes	1.23	0.86	1.76	0.25				
	No	1.00							
t(4;14)	Yes	1.44	0.91	2.26	0.12				
	No	1.00							
Del(17p)	Yes	1.76	1.15	2.70	0.009	1.63	1.02	2.60	0.04
	No	1.00							
Risk stratification	Standard risk	1.00							
	High risk	2.04	1.54	2.69	<0.001				

Supplementary Table S5. Regression analysis for factors associated with overall survival (OS).

Factors		Univariable analysis				Multivariable analysis			
		**HR**	**95% CI**		***P***	**RR**	**95% CI**		***P***
	**IP**	1.26	0.88	1.78	0.21	0.93	0.61	1.41	0.72
	**Age at diagnosis**	0.99	0.97	1.02	0.62				
	**Number of cells infused**	1.06	0.99	1.14	0.08	1.03	0.94	1.12	0.52
**Gender**	Male	1.00				1.00			
	Female	0.71	0.50	1.01	0.05	0.71	0.46	1.10	0.13
**Race**	Non-Hispanic White	1.00				1.00			
	Non-Hispanic Black	0.56	0.31	1.04	0.07	0.92	0.46	1.86	0.82
	Other	NA				NA			
**MM type**	IGA	1.00				1.00			
	IGG	0.67	0.45	1.00	0.049	0.73	0.44	1.21	0.22
	Light Chain only	0.76	0.48	1.21	0.25	0.58	0.31	1.08	0.09
**MM ISS stage**	I	1.00				1.00			
	II	1.23	0.80	1.87	0.34	1.05	0.64	1.71	0.86
	III	1.87	1.24	2.83	<0.001	1.68	1.03	2.74	0.038
**Post-induction response**	CR	0.69	0.41	1.16	0.162				
	VGPR	0.65	0.40	1.08	0.10				
	PR	0.68	0.42	1.08	0.10				
	SD/MR/PD	1.00							
**Pre-transplant response**	CR	0.70	0.43	1.14	0.150				
	VGPR	0.61	0.37	1.02	0.06				
	PR	0.89	0.58	1.38	0.61				
	SD/MR/PD	1.00							
**Conditioning regimen**	Melphalan 140 (mg/m^2)	1.00				1.00			
	Melphalan 200 (mg/m^2)	0.67	0.43	01.03	0.07	0.90	0.52	1.57	0.72
**Maintenance post-transplant**	No	1.00				1.00			
	Yes	0.04	0.02	0.09	<0.001	0.05	0.02	0.12	<0.001
**Cytogenetics**									
1q21+	Yes	1.88	1.30	2.72	0.001	1.71	1.12	2.61	0.01
	No	1.00				1.00			
t(11;14)	Yes	1.00	0.61	1.65	1.00				
	No	1.00							
t(4;14)	Yes	1.18	0.64	2.20	0.60				
	No	1.00							
Del(17p)	Yes	2.17	1.30	3.62	0.003	1.81	1.03	3.18	0.04
	No	1.00				1.00			
Risk stratification	Standard risk	1.00							
	High risk	2.11	1.47	3.03	<0.001				

Abbreviations: HR – Hazard ratio, CI - confidence interval, MM - multiple myeloma, ISS - International staging system, CR - complete response, VGPR - very good partial response, PR - partial response, MR - minimal response, SD - stable disease, *P* – p-value.

Supplementary Table S6. Patient characteristics in the cohort of patients treated with VRD induction.

Variable of Interest	All (n = 165)		NP (n = 126)		IP (n = 39)		*P*
**Age at diagnosis, median (range)**	57	(39-72)	57	(39-72)	57	39-71	0.49
**Number of cells infused, median (range)**	3.97	2.01-15.55	3.93	2.01-15.55	4.22	2.01-9.03	0.480
**Gender (no, %)**							0.53
**Male**	103	62.4	77	61.1	26	66.7	
**Female**	62	37.6	49	38.9	13	33.3	
**Race (no, %)**							0.23
**NHB**	23	13.9	19	15.1	4	10.3	
**NHW**	141	85.5	107	84.9	34	87.2	
**Other**	1	0.6	0	0.0	1	2.6	
**MM type (no, %)**							0.13
**IGA**	36	21.8	32	25.4	4	10.3	
**IGG**	94	57.0	68	54.0	26	66.7	
**Light chain disease**	35	21.2	26	20.6	9	23.1	
**MM ISS stage (no, %)**							0.42
**I**	58	40.3	45	41.3	13	37.1	
**II**	45	31.3	31	28.4	14	40.0	
**II**	41	28.5	33	30.3	8	22.9	
**Conditioning regimen (no, %)**							0.88
**Melphalan 140 (mg/m^2)**	20	12.1	15	11.9	5	12.8	
**Melphalan 200 (mg/m^2)**	145	87.9	111	88.1	34	87.2	
**Maintenance post- ASCT (no, %)**							0.33
**No**	22	13.3	15	11.9	7	17.9	
**Yes**	143	86.7	111	88.1	32	82.1	
**Cytogenetics**	**n = 141**		**n = 110**		**n = 31**		
**1q21+**							0.58
**No**	99	70.2	76	69.1	23	74.2	
**Yes**	42	29.8	34	30.9	8	25.8	
**t(11;14)**							0.14
**No**	117	83.0	94	85.5	23	74.2	
**Yes**	24	17.0	16	14.5	8	25.8	
**t(4;14)**							0.99
**No**	126	89.4	98	89.1	28	90.3	
**Yes**	15	10.6	12	10.9	3	9.7	
**Del(17p)**							0.36
**No**	124	87.9	95	86.4	29	93.5	
**Yes**	17	12.1	15	13.6	2	6.5	
**High-Risk cytogenetic features**							0.52
**None**	78	55.3	58	52.7	20	64.5	
**One Hit**	50	35.5	42	38.2	8	25.8	
**Double Hit**	11	7.8	8	7.3	3	9.7	
**Triple Hit**	2	1.4	2	1.8	0	0.0	
**Risk stratification**							0.24

**Standard risk**	92	65.2	69	62.7	23	74.2	
**High risk**	49	34.8	41	37.3	8	25.8	
**Post induction response**							0.37
**SD/MR/PD**	17	10.3	15	11.9	2	5.1	
**CR/VGPR/PR**	148	89.7	111	88.1	37	94.9	
**CR**	39	23.6	36	28.6	3	7.7	0.01
**VGPR**	50	30.3	37	29.4	13	33.3	
**PR**	59	35.8	38	30.3	21	53.9	
**SD/MR/PD**	17	10.3	15	11.9	2	5.1	
**Pre-transplant response**							
**SD/MR/PD**	21	12.7	13	10.3	8	20.5	0.10
**CR/VGPR/PR**	144	87.3	113	89.7	31	79.5	
**CR**	44	26.7	44	34.9	0	0.0	<0.001
**VGPR**	45	27.3	37	29.4	8	20.5	
**PR**	55	33.3	32	25.4	23	59.0	
**SD/MR/PD**	21	12.7	13	10.3	8	20.5	
**Post-transplant response**							
**SD/MR/PD**	6	3.7	4	3.2	2	5.3	0.63
**CR/VGPR/PR**	156	96.3	120	96.8	36	94.7	
**CR**	78	48.1	68	54.9	10	26.3	0.01
**VGPR**	44	27.2	31	25.0	13	34.2	
**PR**	34	21.0	21	16.9	13	34.2	
**SD/MR/PD**	6	3.7	4	3.2	2	5.3	

Abbreviations: MM - multiple myeloma, VRD – Bortezomib, lenalidomide, dexamethasone, ISS - International staging system, ASCT - autologous stem cell transplant, *P* – p-value.

Supplementary Table S7. Regression analysis for factors associated with progression-free survival (PFS) in patients treated with VRD induction.

Factors		Univariable analysis				Multivariable analysis			
		**HR**	**95% CI**		***P***	**RR**	**95% CI**		***P***
	**IP**	2.02	1.31	3.12	0.002	1.96	1.17	3.29	0.01
	**Age at diagnosis**	0.98	0.96	1.01	0.26				
	**Number of cells infused**	1.13	1.04	1.23	0.01	1.12	1.01	1.24	0.03
**Gender**	Male	1.00							
	Female	0.80	0.53	1.21	0.29				
**Race**	Non-Hispanic White	1.00				1.00			
	Non-Hispanic Black	0.68	0.37	1.28	0.24	0.86	0.42	1.80	0.70
	Other	6.24	0.84	46.22	0.07	2.20	0.27	18.07	0.46
**MM type**	IGA	1.00							
	IGG	0.71	0.44	1.14	0.15				
	Light Chain disease	0.71	0.39	1.29	0.26				
**MM ISS stage**	I	1.00				1.00			
	II	1.22	0.73	2.04	0.45	1.31	0.75	2.27	0.34
	III	1.81	1.09	2.99	0.02	1.71	0.99	2.94	0.06
**Post-induction response**	CR	0.33	0.16	0.65	0.001				
	VGPR	0.39	0.21	0.73	<0.001				
	PR	0.39	0.21	0.72	<0.001				
	SD/MR/PD	1.00							
**Pre-transplant response**	CR	0.32	0.17	0.60	<0.001				
	VGPR	0.31	0.17	0.57	<0.001				
	PR	0.40	0.23	0.72	<0.001				
	SD/MR/PD	1.00							
**Conditioning regimen**	Melphalan 140 (mg/m^2)	1.00							
	Melphalan 200 (mg/m^2)	0.29	0.20	0.44	<0.001				
**Maintenance post-transplant**	No	1.00				1.00			
	Yes	0.29	0.20	0.44	<0.001	0.30	0.19	0.49	<0.001
**Cytogenetics**									
1q21+	Yes	1.41	0.90	2.20	0.136				
	No	1.00							
t(11;14)	Yes	1.28	0.75	2.17	0.36				
	No	1.00							
t(4;14)	Yes	1.81	0.96	3.42	0.07				
	No	1.00							
Del(17p)	Yes	1.40	0.76	2.58	0.277				
	No	1.00							
**Risk stratification**	Standard risk	1.00				1.00			
	High risk	1.96	1.28	3.01	<0.001	2.79	1.72	4.53	<0.001

Abbreviations: VRD – Bortezomib, lenalidomide, dexamethasone, HR – Hazard ratio, CI - confidence interval, MM - multiple myeloma, ISS - International staging system, CR - complete response, VGPR - very good partial response, PR - partial response, MR - minimal response, SD - stable disease, *P* – p-value.

Supplementary Table S8. Regression analysis for factors associated with overall survival (OS) in patients treated with VRD induction.

**Factors**		**Univariable analysis**				**Multivariable analysis**			
		**HR**	**95% CI**		***P***	**RR**	**95% CI**		***P***
**n =165**	**IP**	1.88	1.06	3.33	0.03	1.61	0.81	3.18	0.17
	**Age at diagnosis**	0.96	0.93	1.00	0.06	0.97	0.92	1.01	0.17
	**Number of cells infused**	1.09	0.96	1.24	0.16				
**Gender**	Male	1.00				1.00			
	Female	0.59	0.32	1.09	0.09	0.76	0.33	1.74	0.51
**Race**	Non-Hispanic White	1.00							
	Non-Hispanic Black	0.65	0.26	1.63	0.36				
	Other								
**MM type**	IGA	1.00				1.00			
	IGG	0.54	0.28	1.04	0.065	0.40	0.17	0.95	0.04
	Light Chain disease	0.75	0.35	1.61	0.46	0.61	0.21	1.77	0.36
**MM ISS stage**	I	1.00				1.00			
	II	1.36	0.65	2.85	0.42	1.08	0.47	2.49	0.86
	III	3.11	1.57	6.15	<0.001	2.58	1.14	5.84	0.023
**Post-induction response**	CR	0.33	0.14	0.74	0.007				
	VGPR	0.28	0.13	0.61	<0.001				
	PR	0.28	0.13	0.59	<0.001				
	SD/MR/PD	1.00							
**Pre-transplant response**	CR	0.37	0.17	0.81	0.013				
	VGPR	0.29	0.13	0.66	<0.001				
	PR	0.40	0.20	0.83	0.01				
	SD/MR/PD	1.00							
**Conditioning regimen**	Melphalan 140 (mg/m^2)	1.00				1.00			
	Melphalan 200 (mg/m^2)	0.51	0.24	1.10	0.08	1.28	0.45	3.62	0.64
**Maintenance post-transplant**	No					1.00			
	Yes	0.02	0.00	0.12	<0.001	0.02	0.003	0.17	<0.001
**Cytogenetics**									
1q21+	Yes	1.16	0.62	2.17	0.641				
	No	1.00							
t(11;14)	Yes	1.09	0.53	2.26	0.81				
	No	1.00							
t(4;14)	Yes	1.65	0.64	4.21	0.30				
	No	1.00							
Del(17p)	Yes	2.90	1.44	5.87	0.003				
	No	1.00							
Risk stratification	Standard risk	1.00				1.00			
	High risk	2.62	1.46	4.69	<0.001	2.47	1.28	4.75	0.007

Abbreviations: VRD – Bortezomib, lenalidomide, dexamethasone, HR – Hazard ratio, CI - confidence interval, MM - multiple myeloma, ISS - International staging system, CR - complete response, VGPR - very good partial response, PR - partial response, MR - minimal response, SD - stable disease, *P* – p-value.

Supplementary Table S9. Patient characteristics of samples from non-progressors and patients with interval progression used for flow cytometry analysis.

Variable of interest		All patients (n = 35)	Non progressors (n = 22)	Interval progressors (n = 13)	*P*
**Age at diagnosis**	median (range)	57 (40-71)	56 (40-67)	58 (44-71)	0.29
**Gender (no, %)**	Male	26 (74.2)	18 (81.8)	8 (61.5)	0.24
	Female	9 (25.8)	4 (18.2)	5 (38.5)	
		**n = 31**	**n = 18**	**n = 13**	
**MM ISS Stage (no, %)**					
	I	5 (16.1)	4 (22.2)	5 (50)	0.16
	II	14 (45.2)	6 (33.3)	4 (40)	
	III	12 (38.7)	8 (44.5)	1 (108)	
**Cytogenetic groups (no, %)**		**n = 30**	**n = 17**	**n = 13**	
	Standard-risk	18 (60)	12 (66.7)	10 (76.9)	0.70
	High-risk	12 (40)	6 (33.3)	3 (23.1)	
**Response at time of sample collection (no, %)**	CR/VGPR	13 (37.1)	11 (50)	2 (15.4)	0.07
	PR/MR	22 (62.9)	11 (50)	11 (84.6)	

Figure S1

A

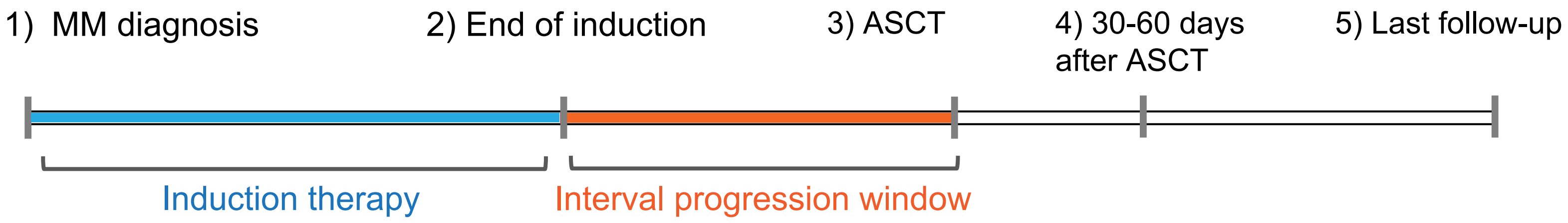

B

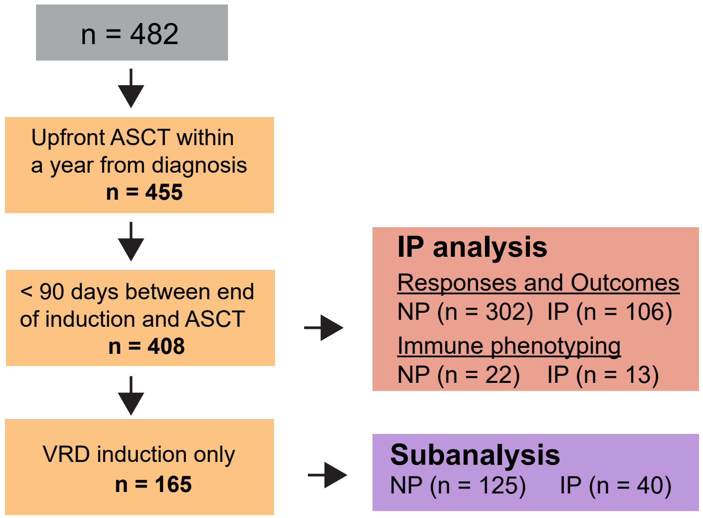

Figure S1. A. Timeline of data collection. Post-induction responses are evaluated at day of end of induction, pre-transplant responses at Day -2, and post- transplant responses 30-60 days from ASCT. Interval progression is defined at 25% increase in the index protein between the end of induction and the Day -2 of ASCT. MM, Multiple Myeloma; ASCT, Autologous Stem Cell Transplant. **B.** Schema of the retrospective study with different subset analysis. NP, non progressors, IP, patients with interval progression, VRD, bortezomib, lenalidomide, dexamethasone.

Figure S2

A

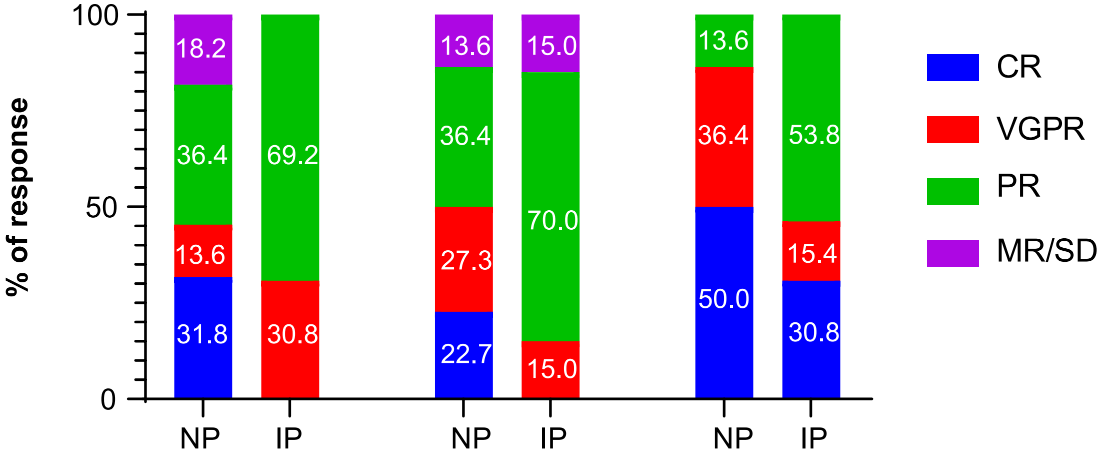

B

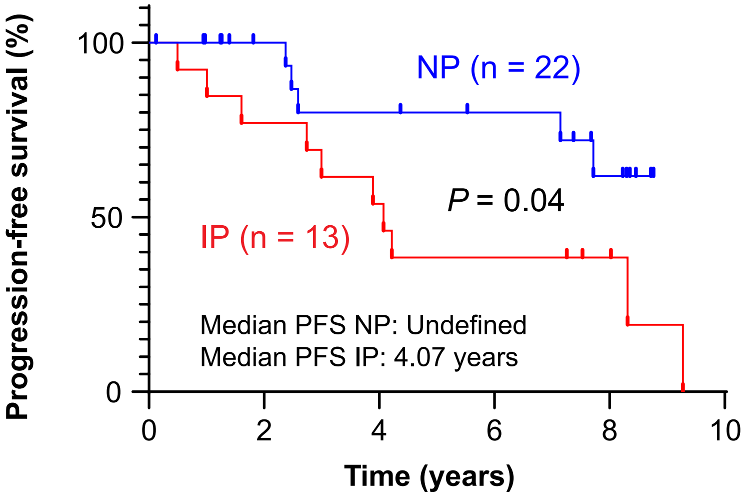

Figure S2. Patients with available samples for flow cytometry staining are representative of the entire cohort. Overall responses (**A**) and progression-free survival from ASCT (**B**) in patients with available peripheral blood samples for immunophenotyping. Log-rank $P = 0.04$.

Figure S3

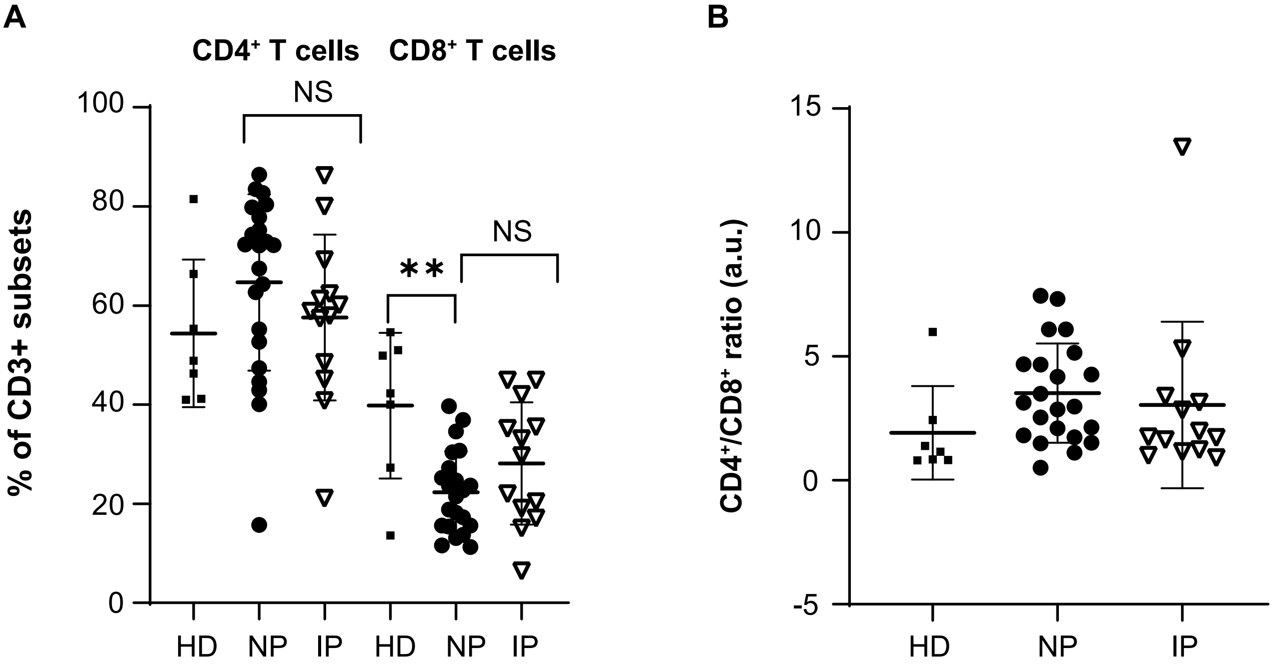

Figure S3. No changes in total CD3$^+$CD4$^+$ T cells, CD3$^+$CD8$^+$ T cells, or CD4$^+$/CD8$^+$ ratios in non-progressors (NP) compared to patients with interval progression (IP). A. Percentages of CD3+, CD4+ T cells or CD3+, CD8+ T cells in 7 healthy donors (HD), 22 NP, and 13 patients with IP. Median with interquartile ranges are shown. CD4+ analysis: One-way ANOVA with Bonferroni's multiple comparison test: NS, HD versus NP; NS, HD versus IP; NS, NP versus IP; ANOVA summary P = 0.2863; CD8+ analysis: One-way ANOVA with Bonferroni's multiple comparison test: **, P = 0.0018, HD versus NP; NS, HD versus IP; NS, NP versus IP; ANOVA summary P = 0.0025. **B.** CD4+/CD8+ ratio as in **A**. One-way ANOVA with Bonferroni's multiple comparison test: NS, HD versus NP; NS, HD versus IP; NS, NP versus IP; ANOVA summary P = 0.3425. Median with interquartile ranges are shown. NS, not significant.

Figure S4

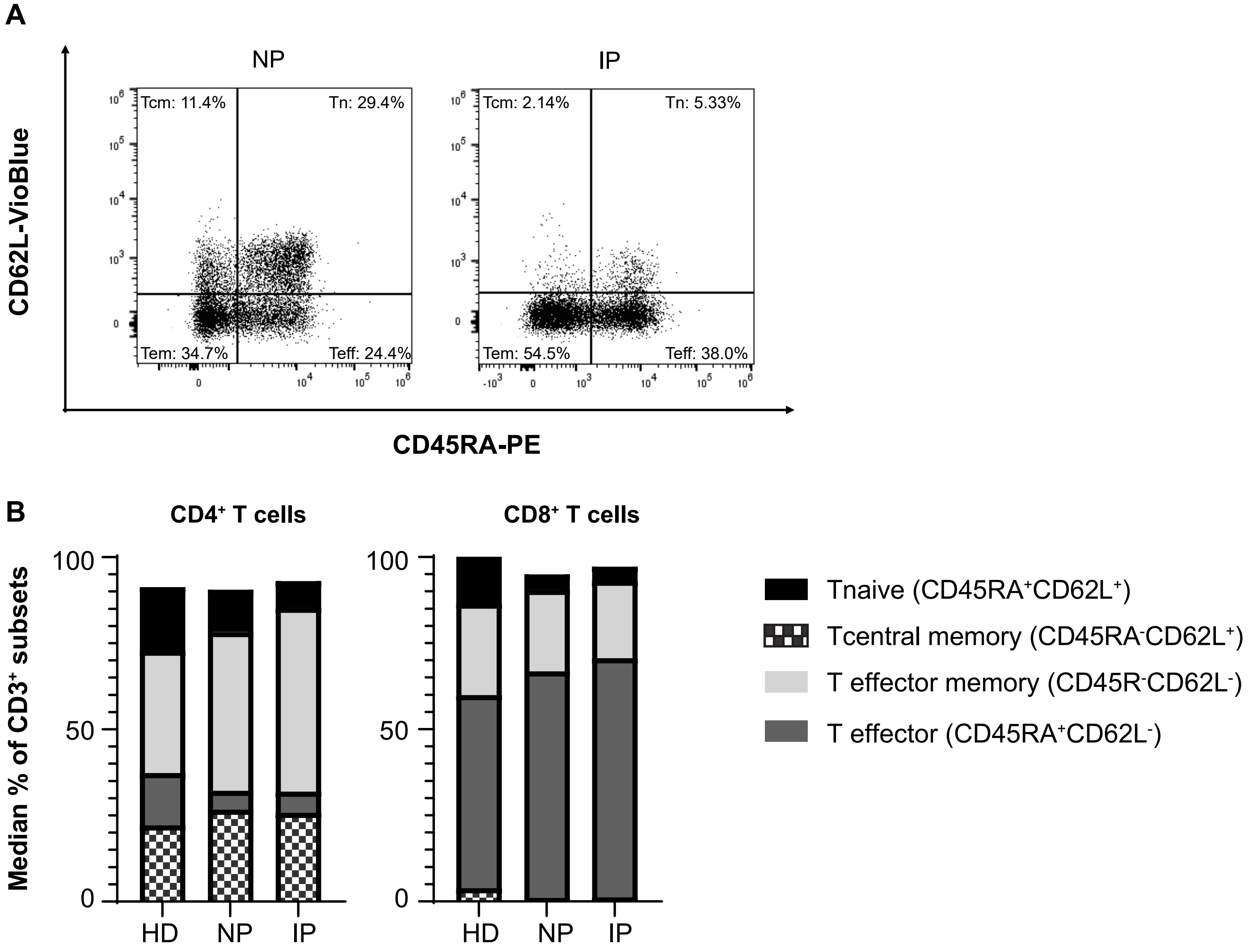

Figure S4. No changes in activation status of $CD4^+$ and $CD8^+$ T cell populations in non-progressors (NP) compared to patients with interval progression (IP). Peripheral blood mononuclear cells (PBMCs) are gated to evaluate $CD3^+CD4^+$ or $CD3^+CD8^+$ T cell subsets. **A.** Dot plot in one representative NP and one representative patient with IP showing the percentage of antigen-exposed effector (Teff: $CD45RA^+$, $CD62L^-$), effector memory (Tem: $CD45RA^-$, $CD62L^-$), central memory (Tcm: $CD45RA^-$,$CD62L^+$), and naïve T cells (Tn: $CD45RA^+$, $CD62L^+$) in the $CD3^+CD4^+$ T cell subset. **B.** Median percentages of the same populations as above in the $CD3^+CD4^+$ T cell subsets or $CD3^+CD8^+$ T cell subsets of 7 healthy donors (HD), 22 NP, and 13 patients with IP.

Figure S5

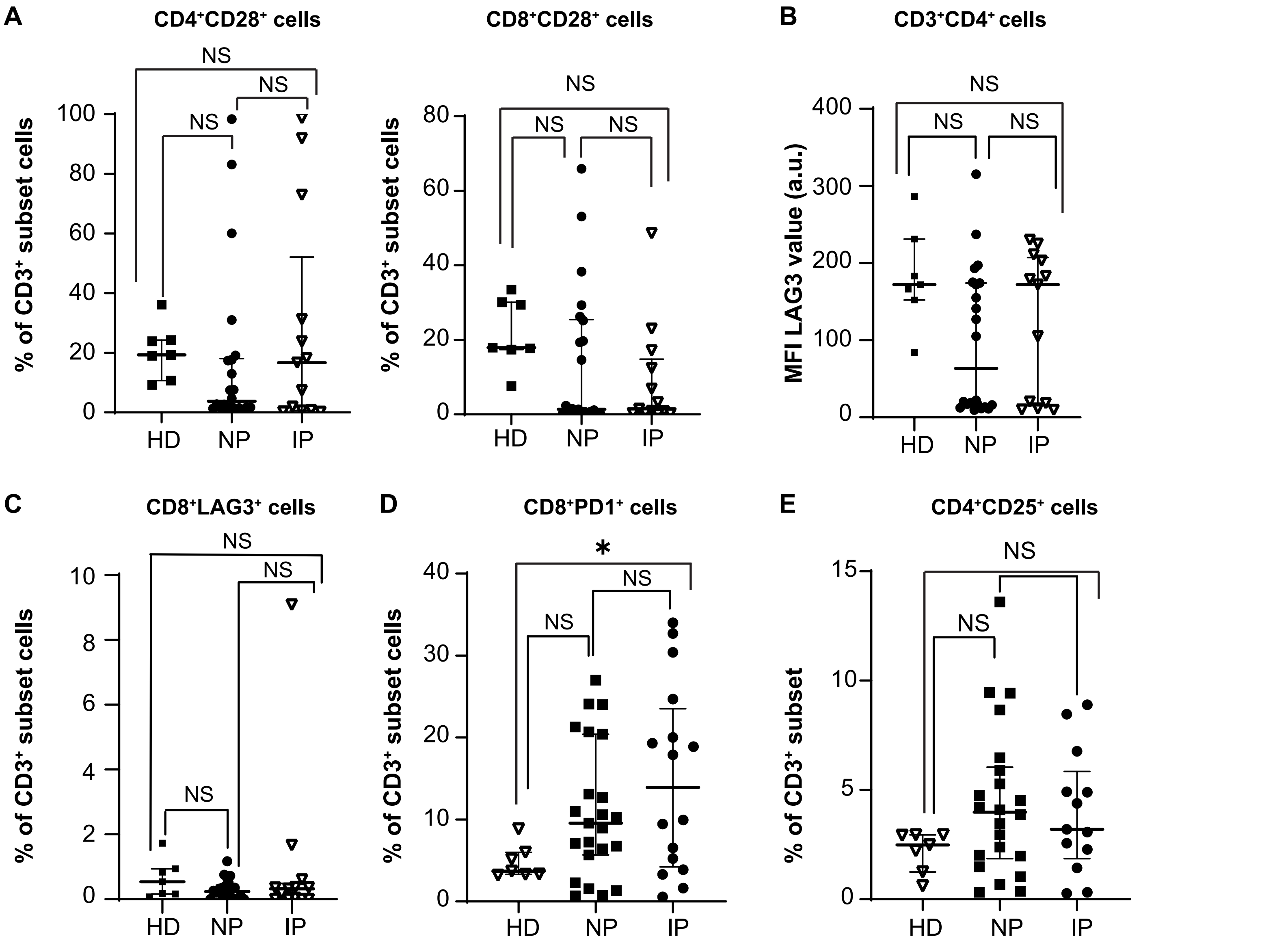

Figure S5. No changes in the percentage or Mean Fluorescence Intensity (MFI) values of other T cell populations in non-progressors (NP) compared to patients with interval progression (IP). Percentage or MFI of the described T cell subsets from 7 healthy donors (HD), 22 NP, and 13 patients with IP are reported in every panel. **A.** Percentage of $CD3^+CD4^+CD28^+$ T cells in HD, NP, and IP. One-way ANOVA with Bonferroni's multiple comparison test: NS, HD versus NP; NS, HD versus IP; NS, NP versus IP, ANOVA Summary *P* = 0.5584 or $CD3^+CD8^+CD28^+$ T cells in ND, NP, and IP. One-way ANOVA with Bonferroni's multiple comparison test: NS, HD versus NP; NS, HD versus IP; NS, NP versus IP, ANOVA Summary *P* = 0.2502. **B.** MFI values of LAG3 in $CD3^+$, $CD4^+$ cells of HD, NP, or IP. One-way ANOVA with Bonferroni's multiple comparison test: NS, HD versus NP; NS, HD versus IP; NS, NP versus IP, ANOVA Summary *P* = 0.1160. Percentages of **C.** $CD3^+CD8^+LAG3^+$ cells in HD, NP, and IP. One-way ANOVA with Bonferroni's multiple comparison test: NS, HD versus NP; NS, HD versus IP; NS, NP versus IP, ANOVA Summary *P* = 0.3713. **D.** $CD3^+CD8^+PD1^+$ cells in HD, NP, and IP. One-way ANOVA with Bonferroni's multiple comparison test: NS, HD versus NP; *, *P* = 0.0492, HD versus IP; NS, NP versus IP, ANOVA Summary *P* = 0.0522. **E.** CD3+CD4+CD25+ cells (T regulatory cells) in HD, NP, and IP. One-way ANOVA with Bonferroni's multiple comparison test: NS, HD versus NP, NS, HD versus IP; NS, NP versus IP, ANOVA Summary *P* = 0.2442.

Supplementary figure legends:

Figure S1. Timeline of data collection. Post-induction responses are evaluated at day of end of induction, pre-transplant responses at Day -2, and post- transplant responses 30-60 days from ASCT. Interval progression is defined at 25% increase in the index protein between the end of induction and the Day -2 of ASCT. MM, Multiple Myeloma; ASCT, Autologous Stem Cell Transplant. **B.** Schema of the retrospective study with different subset analysis. NP, non progressors, IP, patients with interval progression, VRD, bortezomib, lenalidomide, dexamethasone.

Figure S2. Patients with available samples for flow cytometry staining are representative of the entire cohort. Overall responses (**A**) and progression-free survival from ASCT (**B**) in patients with available peripheral blood samples for immunophenotyping. Log-rank $P = 0.04$.

Figure S3. No changes in total $CD3^+CD4^+$ T cells, $CD3^+CD8^+$ T cells, or $CD4^+/CD8^+$ ratios in non-progressors (NP) compared to patients with interval progression (IP). A. Percentages of $CD3^+CD4^+$ T cells or $CD3^+CD8^+$ T cells in 7 healthy donors (HD), 22 NP, and 13 patients with IP. Median with interquartile ranges are shown. $CD4^+$ analysis: One-way ANOVA with Bonferroni's multiple comparison test: NS, HD versus NP; NS, HD versus IP; NS, NP versus IP; ANOVA summary $P = 0.2863$; $CD8^+$ analysis: One-way ANOVA with Bonferroni's multiple comparison test: **, $P = 0.0018$, HD versus NP; NS, HD versus IP; NS, NP versus IP; ANOVA summary $P = 0.0025$. **B.** $CD4^+/CD8^+$ ratio as in **A.** One-way ANOVA with Bonferroni's multiple comparison test: NS, HD versus NP; NS, HD versus IP; NS, NP versus IP; ANOVA summary $P = 0.3425$. Median with interquartile ranges are shown. NS, not significant.

Figure S4. No changes in activation status of $CD4^+$ and $CD8^+$ T cell populations in non-progressors (NP) compared to patients with interval progression (IP). Peripheral blood mononuclear cells (PBMCs) are gated to evaluate $CD3^+CD4^+$ or $CD3^+CD8^+$ T cell subsets. **A.** Dot plot in one representative NP and one representative patient with IP showing the percentage of antigen-exposed effector (Teff: $CD45RA^+CD62L^-$), effector memory (Tem: $CD45RA^-CD62L^-$), central memory (Tcm: $CD45RA^-CD62L^+$), and naïve T cells (Tn: $CD45RA^+CD62L^+$) in the $CD3^+CD4^+$ T cell subset. **B.** Median percentages of the same populations as above in the $CD3^+CD4^+$ T cell subsets or $CD3^+CD8^+$ T cell subsets of 7 healthy donors (HD), 22 NP, and 13 patients with IP.

Figure S5. No changes in the percentage or Mean Fluorescence Intensity (MFI) values of other T cell populations in non-progressors (NP) compared to patients with interval progression (IP). Percentage or MFI of the described T cell subsets from 7 healthy donors (HD), 22 NP, and 13 patients with IP are reported in every panel. **A**. Percentage of $CD3^{+}CD4^{+}CD28^{+}$ T cells in HD, NP, and IP. One-way ANOVA with Bonferroni's multiple comparison test: NS, HD versus NP; NS, HD versus IP; NS, NP versus IP, ANOVA Summary P = 0.5584 or $CD3^{+}CD8^{+}CD28^{+}$ T cells in ND, NP, and IP. One-way ANOVA with Bonferroni's multiple comparison test: NS, HD versus NP*;* NS, HD versus IP; NS, NP versus IP, ANOVA Summary P = 0.2502. **B.** MFI values of LAG3 in CD3+, CD4+ cells of HD, NP, or IP. One-way ANOVA with Bonferroni's multiple comparison test: NS, HD versus NP*;* NS, HD versus IP; NS, NP versus IP, ANOVA Summary P = 0.1160. Percentages of **C**. $CD3^{+}CD8^{+}LAG3^{+}$ cells in HD, NP, and IP. One-way ANOVA with Bonferroni's multiple comparison test: NS, HD versus NP*;* NS, HD versus IP; NS, NP versus IP, ANOVA Summary P = 0.3713. **D.** $CD3^{+}CD8^{+}PD1^{+}$ cells in HD, NP, and IP. One-way ANOVA with Bonferroni's multiple comparison test: NS, HD versus NP*;* *, P = 0.0492, HD versus IP; NS, NP versus IP, ANOVA Summary P = 0.0522. **E.** $CD3^{+}CD4^{+}CD25^{+}$ cells (T regulatory cells) in HD, NP, and IP. One-way ANOVA with Bonferroni's multiple comparison test: NS, HD versus NP, NS, HD versus IP; NS, NP versus IP, ANOVA Summary P = 0.2442.
